# The Global Re-emergence of Syphilis and Antimicrobial Resistance Among Men Who Have Sex With Men (MSM), People Living With Human Immunodeficiency Virus (PLWH), and Newborns: A Narrative Review

**DOI:** 10.7759/cureus.111441

**Published:** 2026-06-24

**Authors:** Sana K Habiya, Amreen Khan, Jaweriya Azeem, Saima Anwar, Naima Kazi, Arfa Shahab

**Affiliations:** 1 Medicine, Indian Institute of Medical Science and Research, Jalna, IND; 2 Public Health, Northeastern Illinois University, Chicago, USA; 3 Preventive Medicine, Independent Researcher, Navi Mumbai, IND; 4 Harris School of Public Policy, University of Chicago, Chicago, USA; 5 Economics, Northeastern Illinois University, Chicago, USA; 6 Preventive Medicine, Independent Researcher, New York, USA; 7 Student Health Services, Northeastern Illinois University, Chicago, USA; 8 Preventive Medicine, Independent Researcher, Windsor, CAN

**Keywords:** antimicrobial resistance (amr), congenital syphilis, men who have sex with other men (msm), people living with hiv (plwh), syphilis

## Abstract

Syphilis incidence continues to rise substantially among men who have sex with men (MSM), people living with HIV (PLWH), newborns, and women of reproductive age, despite preventive measures and available treatments. A major contributing factor is the increasing antimicrobial resistance, which complicates current therapeutic strategies for syphilis. This study aims to support the development of evidence-based, targeted interventions designed to address the specific needs of high-risk populations and to minimize the risk of antimicrobial resistance, while contributing to the overall reduction of syphilis infections. To expand our analysis, we obtained data from the Centers for Disease Control and Prevention (CDC) spanning 2020-2024 through the National Notifiable Sexually Transmitted Infections Surveillance System in the United States to evaluate the trends in syphilis. We also conducted a comprehensive literature search across multiple databases and identified additional epidemiological evidence.

Our findings indicate that the incidence of syphilis cases is projected to increase in North America, Europe, and Asia. Although there was a significant decrease in cases of primary and secondary syphilis globally in 2024 compared with 2023, there was an increase in late and congenital syphilis cases over a longer period from 2020 to 2024. Furthermore, the complex interaction between HIV and syphilis appears to contribute to a heightened risk, which poses challenges for infection control and effective treatment. The rising trend of syphilis remains a global threat, thus requiring multilevel public health interventions, including strengthening education, increasing routine testing, ensuring appropriate antibiotic prescribing practices, and promoting early treatment to prevent transmission. Furthermore, to address the rising cases of syphilis, the availability of additional treatment options and the development of vaccines are crucial strategies for strengthening public health protection.

## Introduction and background

Syphilis is a chronic sexually transmitted infectious disease caused by *Treponema pallidum (T. pallidum)* [1,2,3,4,5] and is now re-emerging worldwide as a significant public health concern. According to the World Health Organization (WHO), an estimated eight million adults acquired syphilis in 2022 [1]. Syphilis was considered preventable and manageable with the implementation of several public health initiatives, particularly due to the widespread use of penicillin. However, in recent years, an unexpected upward trend has been observed, with an increase in reported syphilis cases, especially among men who have sex with men (MSM) and people living with HIV (PLWH) [1,6]. Several studies have also documented a marked rise in syphilis cases among pregnant women [7,8]. 

Various factors have been identified as contributing to the increasing trend of syphilis cases in these high-risk groups. One of the key drivers of the rise in syphilis is high-risk sexual behaviors, particularly among MSM and PLWH populations, which account for over half of reported syphilis cases in the United States [6]. In addition, congenital syphilis poses a serious threat to infants, with risks ranging from perinatal death to neurological, musculoskeletal, and hematological complications [9,10]. In 2022, the WHO reported that maternal syphilis caused approximately 150,000 early fetal deaths and stillbirths, 70,000 neonatal fatalities, and 115,000 infants born with congenital syphilis worldwide [1].

This rise in syphilis cases is not merely an indication of changing sexual and social practices but also highlights the need to address critical gaps in prevention, diagnosis, and treatment strategies. Syphilis has been treated primarily with penicillin, which remains the first-line therapy frequently used today. However, limited availability and emerging resistance are common challenges [11,12]. Findings over the last few years of macrolide-resistant *T. pallidum* have raised an alarming concern, particularly in settings where penicillin is not readily available or is contraindicated. Since the widespread introduction and appropriate use of penicillin therapy, it was also observed historically that syphilis cases declined significantly.

Although in recent times there has been a resurgence of syphilis, partly due to the emergence of antibiotic-resistant strains of *T. pallidum*. Mutations in *T. pallidum* associated with resistance have been identified in the 23S rRNA gene, specifically the A2058G and A2059G mutations, as well as in the 16S rRNA gene, including substitutions at codons A965T and G1058C. This further complicates treatment, particularly in settings where access to penicillin is limited [13,14]. Other treatment options, such as ceftriaxone, azithromycin, and doxycycline monotherapies, have been shown to achieve serologic cure rates comparable to penicillin and may be considered when penicillin administration is not feasible [12]. However, despite the availability of several therapeutic options, the continued rise and recurrence of syphilis cases in recent years suggest that current treatment strategies have not been effective in mitigating the disease.

Other factors that hinder effective control of the disease and create persistent inequities among high-risk groups include limited access to diagnostic testing, significant social stigma, challenges in partner notification, and inadequate antenatal screening [15]. Studies have found that among MSM populations, multiple sexual partnerships, inconsistent condom use, and sexual contact with unknown partners are key factors contributing to increased transmission. The overlap between syphilis and HIV is also a major public health concern, as co-infection facilitates the rapid acquisition and transmission of both infections and has implications for both individual and population health. For HIV-infected patients, syphilis infection can accelerate HIV disease progression, complicate clinical management, and increase the risk of onward transmission [16]. Pregnant women represent another vulnerable group, where the risk of vertical transmission to newborns is high, resulting in a range of systemic complications and leading to congenital syphilis [17,18].

This narrative review aims to provide an overview of syphilis as a re-emergent infectious disease, with a focus on the mechanisms underlying antibiotic resistance and concerns for susceptible populations. This review will describe the epidemiological patterns associated with the re-emergence of syphilis and examine the clinical and public health implications of HIV co-infection. The review will also highlight current gaps in surveillance, diagnosis, and treatment, and explore novel prevention and control strategies addressing both biomedical and social determinants of health. The general objective is to support the development of evidence-based, targeted interventions tailored to the specific needs of key at-risk populations, to minimize the risk of antibiotic resistance, and to contribute to the overall control and potential elimination of syphilis infection.

## Review

A literature search was conducted to support this narrative review using major databases, primarily PubMed/MEDLINE and Google Scholar, as well as targeted searches of relevant public health agency websites, including the Centers for Disease Control and Prevention (CDC) and the WHO. The search focused on literature related to syphilis epidemiology, antimicrobial resistance in *Treponema pallidum*, HIV co-infection, congenital syphilis, and prevention strategies. Key search terms included combinations of “syphilis,” “*Treponema pallidum*,” “antimicrobial resistance,” “macrolide resistance,” “HIV,” “congenital syphilis,” and “epidemiology.” Articles published in English and relevant to the scope of the review were included. As this is a narrative review, a formal systematic screening process or quality assessment was not conducted.

Global epidemiology of syphilis in vulnerable groups

Syphilis has re-emerged over the past decade as a global public health concern, with rapidly increasing incidence across countries. Primary and secondary syphilis (P&S) rates per 100,000 in the United States have risen from 2.1 per 100,000 in 2001 to 9.5 per 100,000 in 2017, with MSM reporting the highest number of P&S syphilis cases [17,18]. High incidence rates of syphilis were observed in countries across Latin America, Africa, and Asia [19]. The WHO also estimated that seven to eight million new syphilis infections would occur globally in 2022, and that the burden was growing primarily across North America, Europe, and Asia [15,20].

The syphilis epidemic disproportionately impacts high-risk groups such as MSM, HIV-positive persons, sex workers, migrants, and pregnant women. Several of these groups predominantly live in low- and middle-income countries (LMICs), where surveillance and access to care are generally suboptimal or limited [15,16,21,22]. In high-income regions such as Europe and North America, syphilis incidence has increased markedly, with the highest increase among MSM and sexually active urban men. Sub-Saharan Africa and parts of Latin America continue to have high prevalence of syphilis among women of reproductive age and antenatal cases, resulting in persistent rates of congenital syphilis and adverse birth outcomes [15,22,23,24]. An increasing prevalence in Asia among MSM and heterosexual groups, with notably high prevalence in Chinese urban populations and other countries, has also been documented. The rise in syphilis cases was further exacerbated during the COVID-19 pandemic, when there was reduced sexually transmitted infection (STI) testing and increased underreporting of STIs [9,25].

A recent meta-analysis has also shown a syphilis prevalence of 10.4% among MSM, with highly variable regional and country-specific prevalence and significantly higher rates among HIV-positive MSM compared with HIV-negative MSM. The global incidence rate for syphilis among MSM was 76.4 cases per 1,000 person-years [9]. In 2019, WHO also estimated that, on average, 11.8% (range: 5.2-19.6%) of MSM were infected with syphilis across 11 of 25 reporting nations, with more than half of these reporting a prevalence rate above 10%. A prevalence rate below 25% for untreated syphilis would result in serious health complications among infected individuals, with an additional role in accelerating the risk of HIV acquisition and transmission [26].

**Figure 1:**
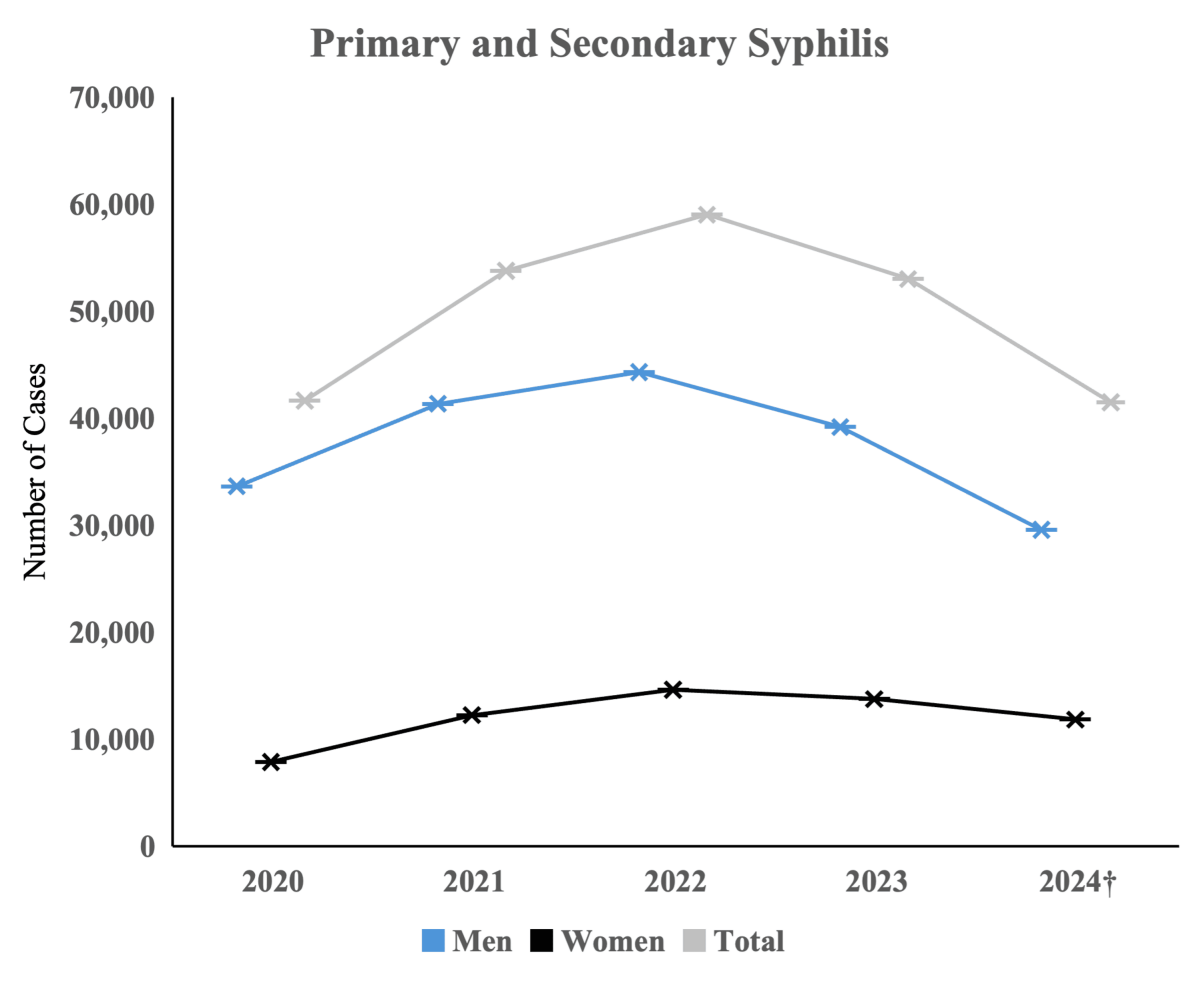
Primary and secondary syphilis cases from 2020 to 2024 Figure created by Jaweriya Azeem in Microsoft Excel using CDC data [27] CDC: The Centers for Disease Control and Prevention

**Figure 2:**
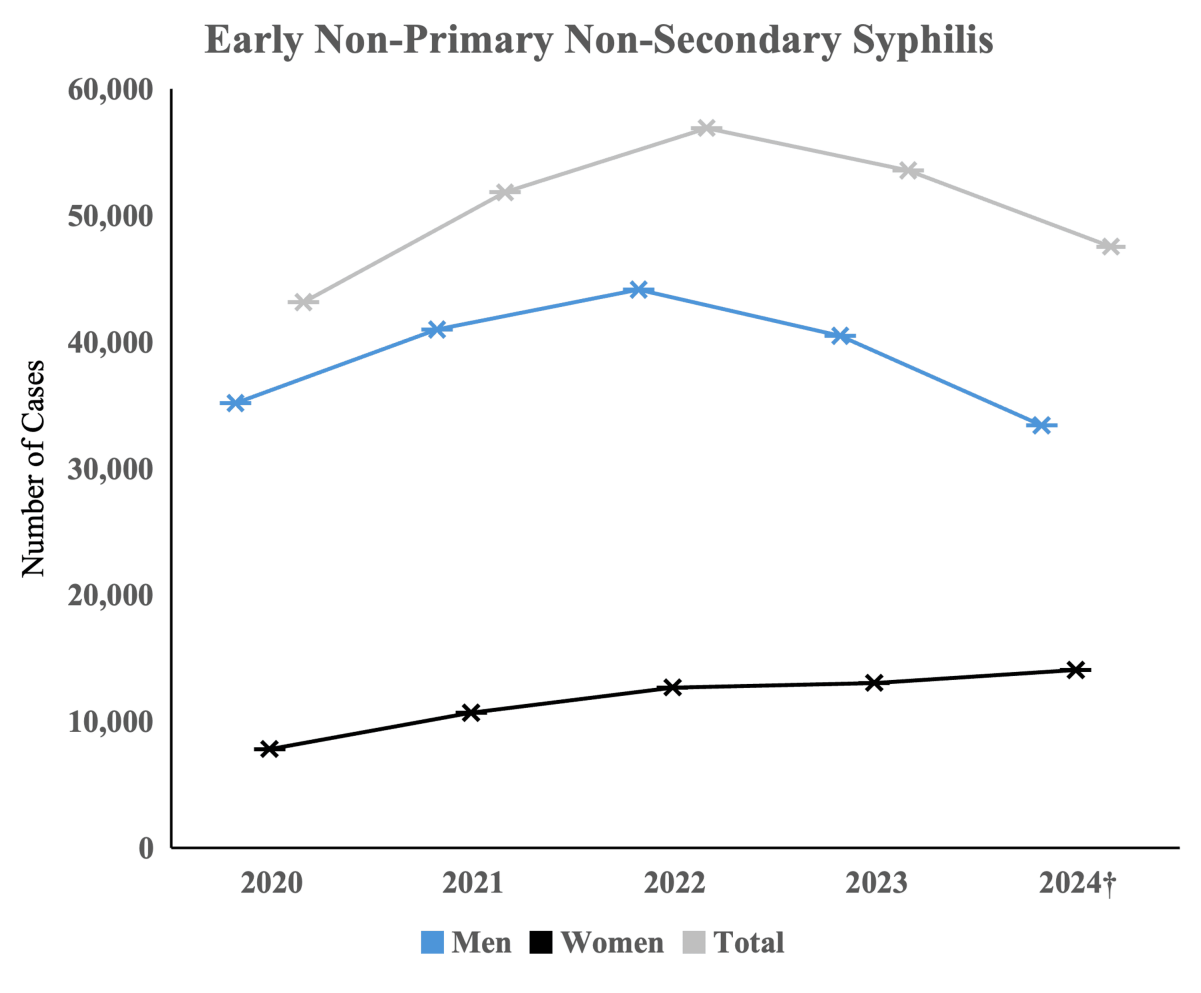
Early non-primary and non-secondary syphilis cases from 2020 to 2024 Figure created by Jaweriya Azeem in Microsoft Excel using CDC data [27] CDC: The Centers for Disease Control and Prevention

**Figure 3:**
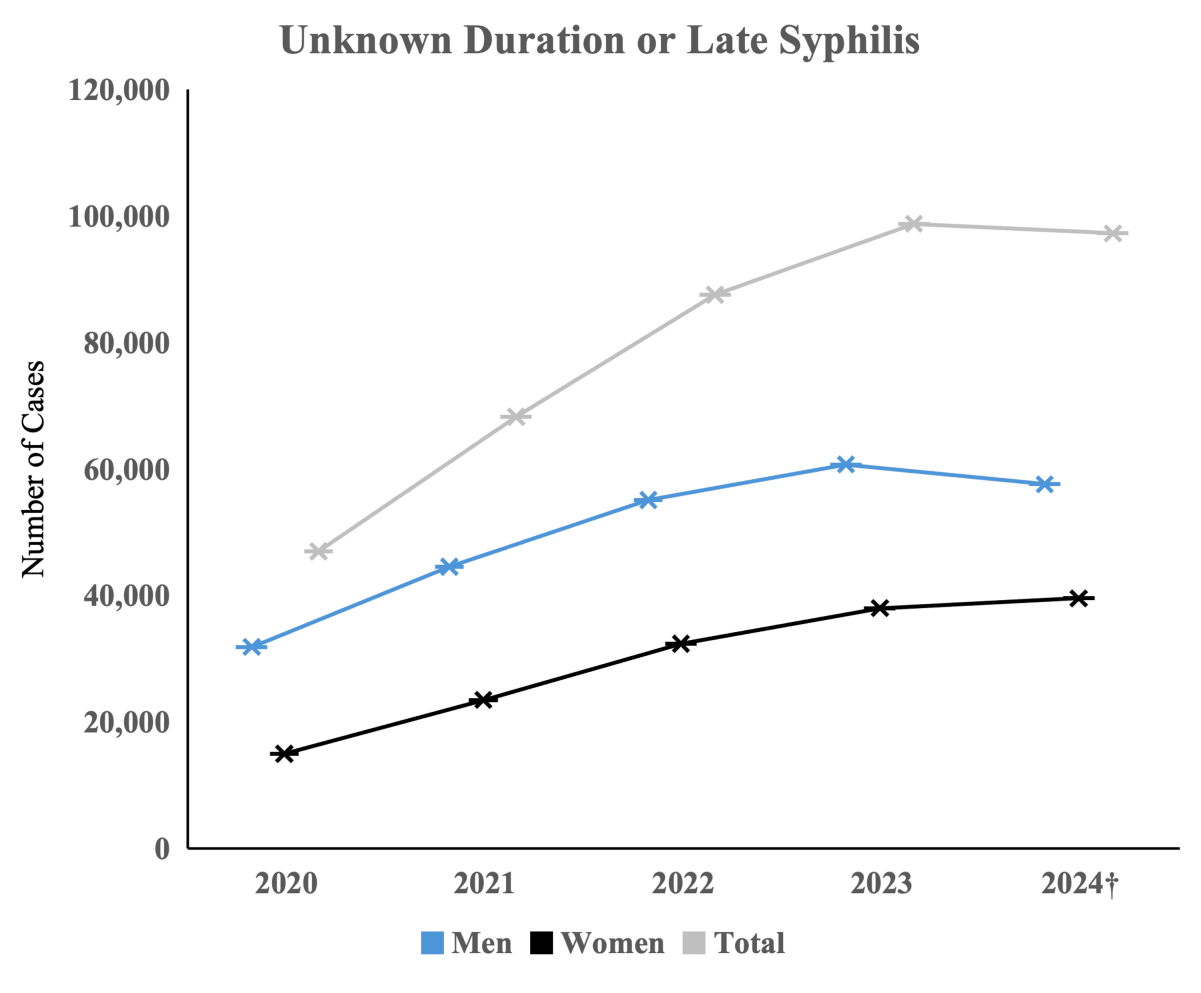
Cases of unknown duration or late syphilis cases from 2020 to 2024 Figure created by Jaweriya Azeem in Microsoft Excel using CDC data [27] CDC: The Centers for Disease Control and Prevention

**Figure 4:**
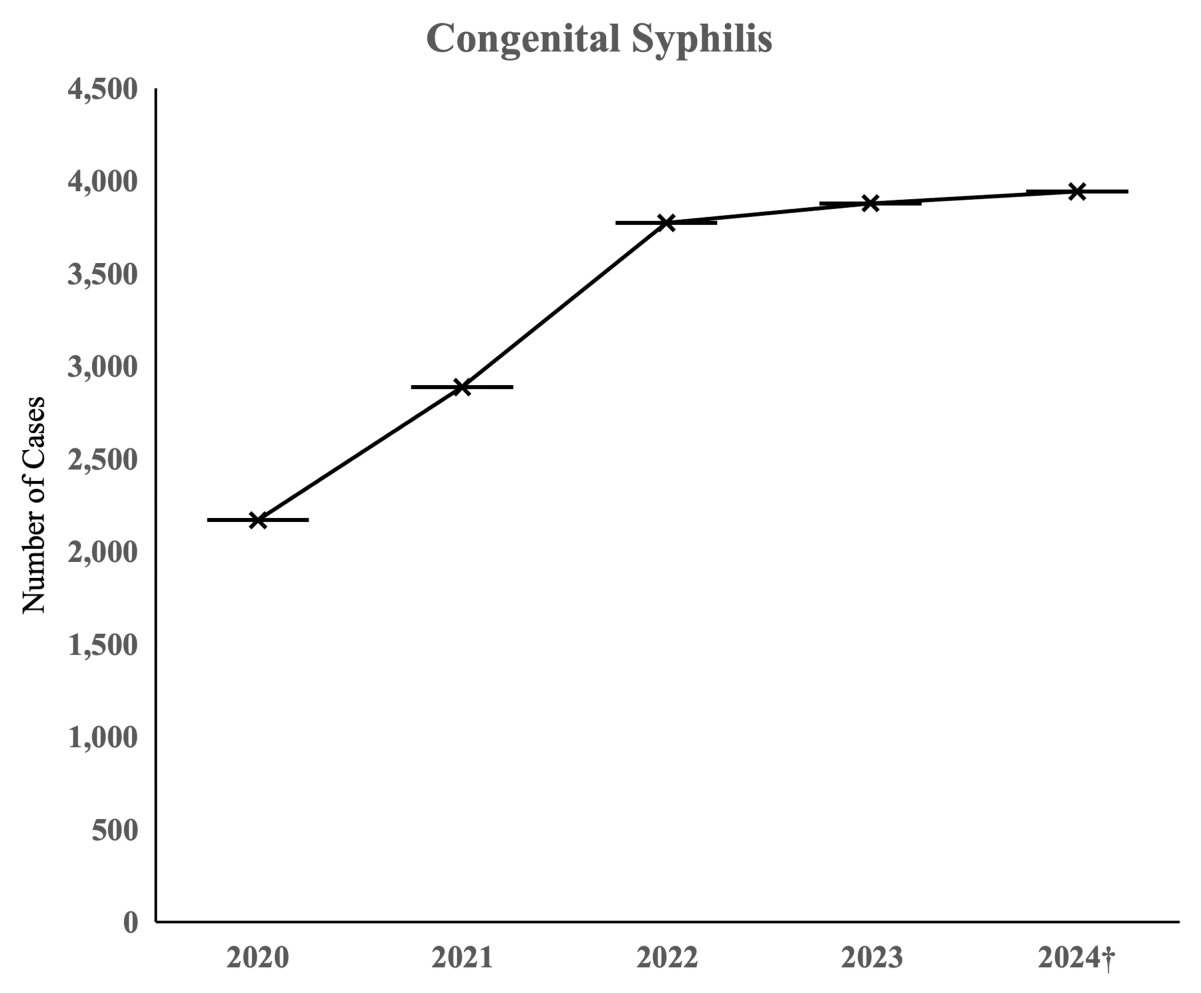
Total number of cases of congenital syphilis from 2020 to 2024 Figure created by Jaweriya Azeem in Microsoft Excel using CDC data [27] CDC: The Centers for Disease Control and Prevention

Data were acquired from the Centers for Disease Control and Prevention (CDC) Nationally Notifiable Sexually Transmitted Infections in the United States from 2020 to 2024, and were used for generating individual graphs on Microsoft Excel as a visual representation of the findings (Table 1). Notably, cases of primary and secondary syphilis have experienced a 22% decrease from 2023 to 2024 (Figure 1) [27]. There was a slight increase in the number of cases in women of early non-primary, non-secondary syphilis from 2020 to 2024 (Figure 2) [27]. An increasing trend in the number of late syphilis cases was observed from 2020 to 2024 (Figure 3). Although the 2024 data is provisional, a consistent increase in reported cases persists. Our findings have shown an increase in syphilis cases in newborns, despite global efforts to prevent it. The CDC reported 4,000 new cases of congenital syphilis in the United States in 2024 alone, and this increase follows a 12-year consecutive trend. This data reveals a rising trend in congenital syphilis cases during this period (Figure 4) [27].

**Table 1:** Nationally Notifiable Sexually Transmitted Infections in the United States from 2020 to 2024 ^*^Total includes cases reported with missing information on sex. ^†^Data are provisional as of August 14, 2025 The data provided in the table were previously published on the CDC website [27] CDC: The Centers for Disease Control and Prevention

Disease	Sex^*^	Cases					Percentage change	
		2020	2021	2022	2023	2024^†^	5-year	1-year
Total syphilis	Total	133,965	176,744	207,273	209,249	190,242	42	-9.1
Congenital syphilis	Total	2,168	2,886	3,773	3,878	3,941	81.8	1.6
Primary and secondary syphilis	Men	33,646	41,349	44,309	39,188	29,575	-12.1	-24.5
	Women	7,901	12,265	14,652	13,763	11,859	50.1	-13.8
	Total	41,655	53,767	59,016	53,007	41,496	-0.4	-21.7
Early non-primary non-secondary syphilis	Men	35,165	40,979	44,143	40,486	33,401	-5	-17.5
	Women	7,809	10,668	12,674	13,036	14,061	80.1	7.9
	Total	43,145	51,830	56,913	53,573	47,539	10.2	-11.3
Unknown duration or late syphilis	Men	31,868	44,548	55,094	60,718	57,591	80.7	-5.2
	Women	14,959	23,474	32,347	37,996	39,540	164.3	4.1
	Total	46,997	68,261	87,571	98,791	97,266	107	-1.5

Current status of antimicrobial resistance in syphilis

Antimicrobial resistance (AMR) represents an additional layer of complexity. The economic and health consequences of AMR are substantial. Drug-resistant infections currently result in an estimated 700,000 deaths annually across the globe. It is projected to reach 10 million deaths by 2050, costing up to US$ 100 trillion worldwide if no action is taken [28]. Resistance to macrolides, which were the first alternatives to penicillins for treating *T. pallidum* infections, emerged in the 1960s. Treatment failure of syphilis with erythromycin was first reported as early as 1964 [29]. Resistance was later reported for azithromycin, clindamycin, and rifampin. Despite growing concern regarding potential resistance to tetracyclines and their derivatives, current evidence supporting this trend remains limited [29].

Macrolide-resistant syphilis is present across a broad geographic range, including Australia, Canada, China, Europe, and the USA [30]. Higher antibiotic use has been reported in several countries, particularly in LMICs, due to limited control of antimicrobial resistance [28]. The distribution of syphilis also varies between LMICs and high-income countries; LMICs still have an endemic prevalence in their overall populations. LMICs typically have a higher syphilis burden, and the proportion of LMICs treating more than 75% of their diagnosed cases is lower compared with high-income nations [30]. Meanwhile, high-income nations have focused on syphilis epidemics among particular groups such as MSM, transgender women, sex workers (SWs), and injecting drug users [30,31]. Nevertheless, MSM, transgender women, and SWs consistently bear a disproportionate burden of syphilis across all regions [30].

Despite these challenges, *T. pallidum* remains highly sensitive to penicillin, which continues to serve as the cornerstone of treatment across all stages of syphilis. However, clinically significant resistance to macrolides has been attributed to point mutations within the 23S rRNA gene [14]. The expanding use of alternative antibiotics raises concerns regarding the emergence of additional resistance mechanisms, particularly those associated with mutations in ribosomal RNA genes. Furthermore, findings from an experimental study provide evidence of the existence of a specific mutation in one of the penicillin-binding proteins, A1873G in the TP0705 gene [32]. This identifies a definitive genetic basis for resistance of syphilis bacteria to the aforementioned β-lactam antibiotics and, in addition, suggests that this mutation is already prevalent among modern SS14 strains [32]. The mutations that have the potential to spread are concerning, as they may contribute to treatment failure and complicate disease control efforts.

Collectively, these findings underscore the urgent need for enhanced surveillance, improved diagnostic strategies, and the development of novel therapeutic approaches, including the pursuit of an effective syphilis vaccine.

HIV co-infection with syphilis

Syphilis and HIV share a synergistic relationship. Syphilis can increase the acquisition and transmission of HIV, and conversely, HIV can affect the manifestation, testing, and course of syphilis, making co-infection a serious and rapidly emerging public health concern [33]. In individuals living with HIV, serological testing for syphilis may be less reliable, with both false-negative and false-positive results reported, thereby complicating diagnosis and clinical management [34]. In advanced HIV infection, treponemal tests such as the *T. pallidum* hemagglutination assay (TPHA) and fluorescent treponemal antibody absorption test (FTA-ABS) may show declining antibody titers and can occasionally become non-reactive. Consequently, a negative result does not necessarily exclude prior or latent syphilis infection in this population [35].

In addition, the prozone phenomenon, characterized by false-negative results in non-treponemal tests due to excessively high antibody titers, has also been noted with greater frequency among patients with HIV and secondary syphilis, indicating that testing must be repeated with dilution when there is a strong clinical suspicion of infection despite negative results [33,36]. Moreover, clinical research suggests that syphilis has tangible effects on markers associated with HIV progression. As seen in observational analyses of data collected among HIV-positive men with primary or secondary syphilis, an average increase in viral load of 0.2 log10 copies per mL during the active phase of syphilis has been reported, followed by a subsequent decrease after appropriate treatment [37].

Epidemiologically, the burden of syphilis among people living with HIV (PLWH) remains substantial. A systematic review and meta-analysis of 29 studies involving 34,740 PLWH in China reported an overall syphilis prevalence of 19.9% (95% CI: 15.4-24.8%), with particularly high prevalence among MSM [38]. Similar findings have been reported in other meta-analyses, with an overall prevalence of 18.6%, further identifying PLWH as a key high-risk population and emphasizing the need for integrated prevention and control strategies targeting both infections [39]. With the advent of the global SARS-CoV-2 pandemic, care for PLWH and syphilis was further affected by administrative and logistical barriers to diagnostic testing, due to reduced access to general health services resulting from lockdowns and social distancing measures.

This implies an increase in data management and reporting errors at large and further worsens access to treatment and testing [39]. Although an overall improvement has been observed in survival and quality-of-life outcomes for PLWH due to universal access to appropriate antiretroviral therapy, changes in sexual practices in these groups have also been observed. This is largely due to reduced adherence to consistent condom use, which has been reported to facilitate syphilis transmission within high-risk populations, including PLWH [37]. Overall, the intersection of HIV and syphilis underscores the need for integrated screening programs, improved diagnostic strategies, and targeted public health interventions to effectively address this dual burden.

Surveillance, prevention, and control strategies

To better understand the patterns of transmission and disease clusters, several interventions are effective in controlling the disease. One such method includes genomic surveillance, which can play a significant role in understanding transmission chains and informing the prioritization of public health interventions [40,41]. Another intervention includes community education initiatives, which have also proven to be an effective method in changing attitudes and knowledge about safe sex practices, regular screening, antibiotic use, and antimicrobial resistance [28]. Implementing a policy that supports national efforts to promote awareness of responsible antibiotic use can also play a major role in the prevention of antibiotic resistance cases [28]. 

Providing education at all levels (community, healthcare, and individual) is essential to the control and prevention of syphilis [28]. Education should include information on detecting clinical signs, identifying people at highest risk based on local epidemiology, and screening, particularly for pregnant women early in pregnancy, again at 28-32 weeks’ gestation, and at delivery [42]. Patients and the general public should be educated, particularly early in an epidemic, so that they may understand the risks, recognize rashes and painless ulcers, avoid high-risk behaviors, seek screening early in pregnancy, and encourage partner notification. Messages can take the form of television and radio spots, posters, booklets, internet banner ads, websites, message boards, online chats, and community meetings, depending on who is most vulnerable and where they can be reached [43]. 

Public health professionals can also work with institutions that serve people at high risk of contracting syphilis, such as drug treatment centers, jails/prisons, local MSM community health organizations, and maternal-child health programs [17]. The US Preventive Services Task Force strongly advises syphilis screening (grade A) for anyone who is at high risk for the disease. In most regions, MSM and PLWH are at a higher risk currently. Interventions to increase screening in clinical settings have been more effective when including automatic testing as part of a routine visit or patient reminders for screening [43]. In locations with high syphilis incidence among MSM, routine annual screening should be promoted in MSM-focused clinics [43,44]. Inclusion of Pre-exposure prophylaxis (PrEP) is highly effective as one of the game-changing tools for prevention of the disease. By integrating PrEP programs in healthcare services, access gaps can be closed, improve user adherence, and enhance syphilis as well as other STI prevention, creating a crucial opportunity for better comprehensive sexual health care [45].

Changes in behavior may additionally lower the risk of syphilis. In the 1980s, condom use surged while syphilis rates fell among MSM, owing in part to AIDS-related concerns. In a randomized controlled trial conducted early in the AIDS epidemic, STD and HIV prevention counseling reduced the probability of STDs by 20% over 12 months (from 14.6% to 11.5%) [46]. Community-level initiatives have a broad impact; however, they are difficult to evaluate. A recent study of community efforts to encourage condom use indicated modest increases in condom use but no statistically significant reduction in STDs [42].

Major health departments in the United States employ trained professionals who assist patients in notifying their partners [43]. Because locating partners can be challenging, partner notification strategies have expanded beyond traditional in-person methods to include phone calls and other approaches. In Monroe County, New York, investigators used smartphone apps to identify partners for patients who lacked other contact information. This method successfully reached six of 21 partners who had connected online or through an app, with two additional partners notified via a website [43,47]. In North Carolina, individuals who could not be contacted through standard partner notification efforts were referred to a single coordinator, who successfully reached an additional 230 contacts through email and, when unsuccessful, reached another 14 through text messaging [17,43,48]. These efforts led to 13 new cases of syphilis and eight newly diagnosed HIV infections, representing about 13% of all syphilis or HIV notifications that year. Collectively, these findings highlight the effectiveness of incorporating digital communication methods into partner notification strategies.

The fields of public health and medicine have traditionally operated in parallel, with frequently overlapping agendas. Efforts to integrate these two professions through shared and pooled resources will improve STI surveillance, prevention strategies, and treatment outcomes. This relationship has the potential to improve the efficiency of individual services while also benefiting sexual health at the population level [17].

## Conclusions

Considering the evidence presented above, syphilis remains one of the most complex challenges in public health. If not treated or controlled, it can affect many people and lead to serious, life-threatening complications. There is an increase in the incidence of syphilis worldwide despite it being a preventable and curable infection, leading to a considerable burden on public health prevention and control efforts. In addition, the recent occurrence of treatment failures, along with growing concerns about AMR, has further increased concerns about the possibility of antibiotic resistance developing in syphilis cases. Even though penicillin has been used as the primary drug against syphilis due to its effectiveness in treating patients with this condition, there have been some limitations associated with penicillin as the first-line treatment for syphilis. Given the rising prevalence of syphilis in disadvantaged communities around the world, as well as the fact that many newborns are affected by congenital syphilis, there is an even greater need to develop targeted interventions to prevent syphilis cases. The possibility of resistance, while rare, and the limitations of relying on penicillin as the only treatment option are becoming increasingly apparent. Hence, the development of new treatment regimens and vaccines is highly recommended at this stage. Likewise, a wide range of initiatives, partnerships, strategic plans, and coordinated efforts targeting the complex and multidimensional nature of these infections is recommended to prevent and control syphilis. The fight against syphilis must involve multiple stakeholders, new strategies, and interventions, ranging from grassroots initiatives to global alliances.
